# Case report: imaging and treatment of ophthalmic manifestations in oculodentodigital dysplasia

**DOI:** 10.1186/s12886-015-0173-1

**Published:** 2016-01-07

**Authors:** Sameh Mosaed, Bradley H. Jacobsen, Ken Young Lin

**Affiliations:** The Gavin Herbert Eye Institute, University of California, Irvine School of Medicine, 850 Health Sciences Rd, Irvine, CA 92697 USA; University of California, Irvine School of Medicine, 252 Irvine Hall, Irvine, CA 92697 USA

**Keywords:** Chronic angle closure, Oculodentodigital dysplasia, Ciliary body cysts, Ultrasound biomicroscopy

## Abstract

**Background:**

Diagnostic and surgical management of severe chronic angle- closure glaucoma secondary to ciliary body cysts can be difficult to manage in a patient with oculodentodigital dysplasia.

**Case presentation:**

A 6-year old girl with oculodentodigital dysplasia, with progressive chronic angle- closure glaucoma secondary to ciliary body cysts presented to our clinic. The initial examination revealed counting fingers vision in the left eye. Intraocular pressure (IOP), as assessed by tonopen, was 31 mm Hg. Ultrasound biomicroscopy revealed ciliary body cysts in the left eye, and gonioscopy confirmed chronic angle closure. A tube shunt was placed to control the elevated IOP. A year after her tube shunt placement in the left eye, ultrasound biomiscropy was performed on her right eye and showed no ciliary body cysts. Gonioscopy in the right eye revealed an open angle to the ciliary body band. Subsequent serial gonioscopy every 3 months showed gradual narrowing of the right eye angle and finally three-and-a-half years after tube placement of the left eye, her right eye IOP became uncontrolled with medications alone and a tube shunt was similarly placed in the right eye. Intraoperative ultrasound biomicroscopy performed at the time of the right eye tube shunt revealed extensive ciliary body cysts in the right eye. Her IOP in both eyes have been well controlled since the placement of tube shunts.

**Conclusions:**

This is one of the first reported cases of severe chronic angle- closure glaucoma secondary to ciliary body cysts in a patient with oculodentodigital dysplasia. We believe that early screening for ciliary body cysts is important in patients with oculodentodigital dysplasia.

## Background

Oculodentodigital dysplasia is a genetic disorder that impacts the development of the face, eyes, limbs, and teeth [1]. Currently there are very few reported cases of severe chronic angle- closure glaucoma secondary to ciliary body cysts in association with ODDD. Here we report one such example.

## Case presentation

**Fig. 1:**
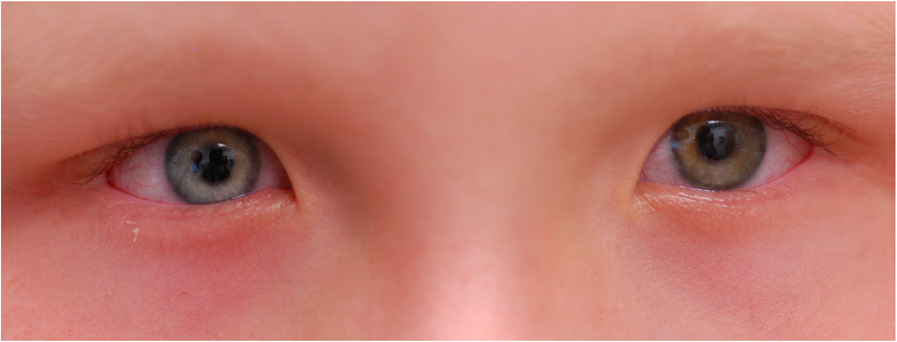
Prominent epicanthic folds

**Fig. 2:**
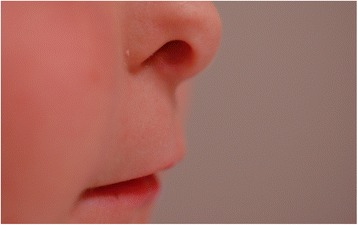
Narrow pinched nose with hypoplastic alae nasi

**Fig. 3:**
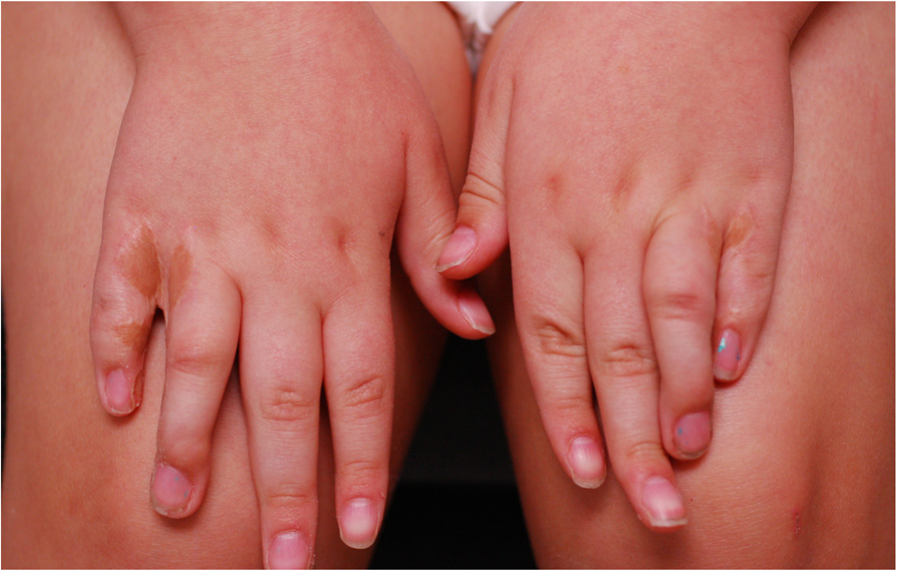
Camptodactyly with evidence of previous syndactyly

A 6-year-old girl with a past medical history significant for oculodentodigital dysplasia syndrome and refractive amblyopia in the left eye was referred for treatment of elevated IOP in the left eye. Visual acuity in the left eye was counting fingers, while vision in the right eye was 20/25 on presentation. Her cycloplegic refraction with retinoscopy was +1.00 + 1.00x180 OD and−7.00 + 2.00x170 OS. Examination under anesthesia (EUA) revealed elevated pressure of 11mmHG OD and 31 mmHg OS by Perkins applanation tonometry. Pressure was checked immediately upon sedating the patient so that the effects of anesthesia on intraocular pressure were minimized. Corneal diameter was 9.5 mm in both horizontal and vertical meridian in the right eye, and 11 mm horizontally by 10.5 mm vertically for the left eye. There was residual tunica vasculosa lentis noted for both eyes. Fundus examination showed a normal sized optic disc in both eyes and a cup-to-disk ratio of 0.1 OD and 0.9 OS. Ultrasound biomicroscopy (UBM) was performed with a 48 MHz probe offering 32 mm field of view, 30 degrees scanning angle, and lateral resolution of 0.05 mm (UBM Plus Guarded, Accutome Inc, Malvern, PA, USA). UBM showed ciliary body cysts only in the left eye. Gonioscopy revealed an open angle OD, but a completely sealed angle OS 360 degrees. The remainder of the intraocular exam was unremarkable. On external exam, the patient has characteristic facial features such as prominent epicanthic folds (Fig. 1) and a narrow pinched nose with hypoplastic alae nasi (Fig. 2). In addition to the facial features, the patient also has camptodactyly with evidence of previous syndactyly (Fig. 3). Due to the advanced stage of glaucoma on presentation and uncontrolled IOP despite timolol 0.5 % and latanoprost 0.005 % application in the left eye, decision was made to proceed with a Baerveldt Glaucoma Implant BG101-350 (Abbott Medical Optics) for her left eye.

The patients’ vision and IOP in both eyes remained stable on timolol 0.5 % and latanoprost 0.005 %. Interval EUAs revealed good position of the Baerveldt glaucoma implant in the left eye. UBM of the right eye was repeated 1 year post Baerveldt Glaucoma Implant of the left eye, and revealed no ciliary body cysts. Gonioscopy of the right eye at that time showed the angle opened to ciliary body band 360°. A repeat gonioscopy of the right eye 2 years post Baerveldt Glaucoma Implant of the left eye, however, showed that the angle now opened to scleral spur temporally and inferiorly, and to only to the posterior trabecular meshwork for the remaining quadrants. During that year her IOP in the right eye began to trend to mid and upper teens. A 24-2 Humphrey automated visual field (Humphrey Visual Field, 24-2 with SITA standard, Zeiss, San Diego, CA) was performed, which despite the generalized depression and high fixation loss, did not show obvious glaucomatous changes in the right eye.

Her left eye IOP and vision remained stable since her initial tube shunt placement, and her right eye IOP ranged between 13 mmHg to 19 mmHg on timolol 0.5 % and latanoprost 0.005 %. However, three-and-a-half-years post glaucoma implant in her left eye, her right eye IOP suddenly was 32 mmHg by applanation, while her vision remained unchanged at 20/25. This IOP elevation was found 5 months after a stable interval visit with acceptable IOP. Fixed- Combination timolol 0.5 %-brimoninidine 1 % was added to her regimen to replace timolol 0.5 %; However, her IOP remained elevated above 30 mmHg on the return visit one week later. An exam under anesthesia with possible Baerveldt glaucoma implant placement was recommended to the patient and parents, given this was her better seeing eye that was now having uncontrolled IOP on maximum tolerated medical therapy. Her IOP by Perkins applanation tonometry during the EUA was 25 mmHg OD and 17 mmHg OS. Her corneal thickness was measured to be 562 microns OD and 620 microns OS. Ultrasound biomicroscopy performed during the procedure revealed ciliary body cysts closing the angle in the right eye (4A–B). Gonioscopy confirmed angle closure with the majority of the angle sealed. A Baerveldt Glaucoma Implant BG101-350 was implanted in the supero-temporal quadrant at that time.

The patient continues to have acceptable IOP in the low teens with retention of poor vision OS, 4 years following implantation of the tube shunt implant in her left eye. Her IOP and vision OD have been stable and acceptable in the low to mid teens for more than 6 months following the aqueous shunt implantation in her right eye with 20/25 best- corrected visual acuity, and a full and reliable automated visual field (Humphrey, 24-2 SITA algorithm). This represents a successful outcome in this complex condition.

## Conclusions

Oculodentodigital dysplasia is a genetic disorder that impacts the development of the face, eyes, limbs, and teeth [1]. It is usually inherited in an autosomal dominant pattern, but has also been identified in sporadic and autosomal recessive cases. Fewer than 1000 individuals have been diagnosed worldwide with ODDD. Genetic mutations in the gap junction alpha 1 gene (GJA1), which code for the transmembrane protein, Connexin are responsible for the defects found in ODDD [3, 5]. Ocular manifestations of ODDD include microophthalmia, microcornea, epicanthus, narrow palpebral fissures, and malformations of the iris, glaucoma, and strabismus [1]. Non-ocular manifestations include cleft lip, cleft palate, low insertion of the columella, thin alae nasi, dental anomalies, syndactyly of the fourth and fifth fingers (seen in 80 % of affected individuals), and neurologic abnormalities. Our patient exhibits the typical facial features of narrow palpebral fissures, epicanthus, low insertion of a prominent columella, cleft lip (repaired) and syndactyly (repaired). However our patient does not have any significant neurologic impairment and indeed is progressing well in school.

**Fig. 4:**
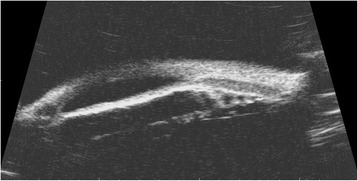
Ultrasound biomicroscopy demonstrating ciliary body cysts in the right eye causing complete angle closure

One rare abnormality observed in patients with ODDD is ciliary body cysts. One study suggests that ciliary body cysts develop due to weakened intercellular adhesions between the ciliary body pigmented and nonpigmented epithelium [2]. Although difficult to visualize, ciliary body cysts can be well documented with ultrasound biomicroscopy, and are the preferred imaging modality when evaluating iris lesions with posterior or ciliary body involvement [6]. Gonioscopy shows narrowing of the angle, but does not demonstrate the cause, and anterior segment optical coherence tomography does not illustrate the ciliary body lesions as well as UBM. Figure 4 demonstrates ciliary body cysts as the mechanism of chronic angle closure in our patient.

This is one of the few reported cases of chronic angle-closure secondary to ciliary body cysts in patients with ODDD. Ciliary body cysts should be considered in the differential for patients with chronic angle closure and/or increased intraocular pressure with ODDD. We were able to visualize our patients’ cysts by utilizing UBM (Fig. 4). Screening patients with ODDD for ciliary body cysts using UBM can be an important diagnostic tool in predicting impending angle closure in these individuals. Serial UBM and frequent examinations are critical in identifying progressive angle closure in these patients and can result in early diagnosis and retention of excellent vision. In a previously reported case of open angle glaucoma associated with ODDD, a trabeculotomy and cyclocryocoagulation were performed to control the pressure [4]. Cyclocryocoagulation is typically reserved for end stage disease given the risks of intraocular inflammation, hypotony, and phthisis. Also, angle-based procedures involving trabecular bypass would be contraindicated in a patient with angle closure. In our patient, trabeculectomy was not considered as trabeculectomies enhanced with antimetabolites carry a 0.2–1.5 % per year lifetime risk of endophthalmitis [7]. This is an unacceptably high risk in a monocular child. Pressures in our patient were successfully controlled with bilateral tube shunts and should be considered in patients with similar presentations.

In summary, ODDD can present with numerous ophthalmic abnormalities such as progressive, severe chronic angle closure, which can be successfully diagnosed with UBM, and treated with aqueous shunt implantation.

## Consent

Written informed consent was obtained from the patients’ parents for publication of this case report and any accompanying images. A copy of the written consent is available for review by the Editor of this journal.

## Abbreviations

IOP: Intraocular pressure
EUA: Examination under anesthesia
UBM: Ultrasound biomicroscopy
GJA1: Gap junction alpha 1 gene
